# Immediate Root Filling

**Published:** 1894-07

**Authors:** Edmond Noyes

**Affiliations:** Chicago.


THE
AMERICAN JOURNAL
—OF—
DENTAL SCIENCE.

VOL. XXVIII. BALTIMORE, JULY, 1894. No. 3.

ARTICLE I.

### IMMEDIATE ROOT FILLING.

DR. EDMOND NOYES, CHICAGO.

A paper on this subject was read before the Illinois State Dental Society, at its last meeting, which discussed very briefly the question "Is immediate root filling advisable:"

1. After heroic extirpation of a live pulp?
2. After removing a devitalized pulp?
3. After removing a putrescent pulp?
4. When there is peridental membrane abscess with fistulous opening?
5. The same without fistulous opening? ("Blind abscess.")

The essay gave an affirmative answer to each proposition. These cases appear to include about all diseased teeth requiring root fillings, except those with acute abscess. Whether the essayist would also fill these at the first sitting did not appear, but it is hard to see why he should not. unless deterred by the illness of the patient or the soreness of the tooth, from making a sitting sufficiently long to accomplish it.

The first two heads, namely, "After extirpation of a live pulp," and "After removing a devitalized pulp," are so nearly alike in their relations to this question that they may be considered together. They furnish the most frequent and suitable opportunities for immediate root fillings, and there are no serious objections to them if required by convenience or necessity. However, when we remember that a large proportion of root fillings have to be made in bicuspids and molars, and consider the difficulty of getting away all portions of the pulps from small roots, we often find that root filling "after the removal of all pulp tissue" does not always prove to be so very "immediate." Probably the recently described methods of cleansing pulp canals by means of kalium-natrium (dilute sulfuric acid), may make the accomplishment of it at one sitting more practicable than heretofore.

The objections to immediate filling after extirpation is complete relate to the difficulty of controlling hemorrhage or exudation, and consequently of obtaining complete dryness of the canals, to the irritability or sensitiveness of the parts at the apex, manifested by severe pain during the introduction of the filling, and which is apt to be much greater if the filling is carried to the apex as it should be, than if it stops short of its proper destination, and lastly the liability of greater soreness and discomfort following the operation, than would be the case if delayed till another sitting.

Often these objections do not exist, or may be easily overcome, and many others in which they may be overcome or endured if it is desirable or necessary. There can be no universal rule. It is likely that country practitioners will complete these operations at once, oftener than those in cities, whose patients are more accessable, and in the habit of being more fully subject to the requirments of their dentists.

Those having putrescent pulps should have a broader definition, if intended to cover those all the way between, but not including those devitalized and filled without sepsis, and those in which an abscess had been formed. The first of the subheads in this class is suppurating pulps; the second, putrescent pulps, and a third, those in which the decomposition and removal of the pulp is complete, but without the formation of an abscess. Each of these three subdivisions implies that the pulp chamber or root canal is open or has been open.

The most significant and important thing about all these cases is sepsis or infection, and there are two decisive objections against immediate root filling, unless for some reason the necessity for it is so urgent as to justify the taking of considerable risk.

The first objection is in the fact that disinfection, to be thorough and trustworthy, requires time for its accomplishment. The second is the impossibility of knowing certainly whether the apical space has been infected, or whether it is infected during the operation, which is liable to happen, notwithstanding all possible care, if the work is completed at the first sitting. A very large proportion of those cases will do well if immediately filled, and some of them are so near like the preceeding ones as really to belong with them. For instance, if a suppurating or putrescent pulp shows some life, and its removal is followed by somewhat free hemorrhage, the portion next the apex appearing to be in a nearly normal condition, there would be little fear of trouble if immediately filled. If, however, the whole root canal appears to be in a septic condition, it cannot be cleaned and filled at once without an unjustifiable risk of subsequent trouble that could propably be avoided by taking more time.

The first point to be gained is the disinfection of the root canals. This is more quickly and surely accomplished after the removal of so much of their contents as can be got away without danger of forcing anything through the apex. Then some strong and diffusible antiseptic should be placed in them for some days. (Free diffusibility is more important than great strength or concentration.

This application may usually be tightly closed in by a gutta-percha stopping, though a vent will sometimes prove necessary. At the second sitting the canals may usually be safely cleaned to the apex, though I have seen some in which oil cassia and myrtol had been tightly enclosed for a week without developing the least trouble, which yet became acutely inflamed, and in some instances formed abscesses, after thorough cleaning and treatment at the second sitting, showing (probably) that the disinfection of the apical portions of the root-canals had not been so complete as was expected. (There may be some cases in which peridental inflammation is caused by the antiseptic instead of by infection, but if that is the cause it will not develop into an abscess.)

The third sub-class above mentioned (in which the decomposition and removal of the pulp are complete), are not always certainly distinguishable from those having blind abscesses, and the same general line of treatment usually proves appropriate in either case, though if there is active formation of pus, or if it becomes active under the stimulation of treatment, a tight closing in of the medicine is, of course, impracticable at first. That anybody should seriously advocate the immediate filling of roots that will not bear tight temporary stopping for half a day without severe pain and inflammation, which is properly relieved by removal of the stopping, seems incomprehensible. Perhaps no one does advocate it in just these cases, but some certainly do in cases that are impossible to distinguish from these at the first sitting. If there is a dead pulp, with the peridental membrane and the tissues at the apical space in a normal condition, we have only to remove the corpse and disinfect the chamber and canals, but if the apical tissues are diseased, we need in addition to have them cured before we fill the roots, or so far in the way of recovery that the remainder of the process can be depended on to complete itself. This would seem to be so reasonable a proposition as to need no argument, but it has been urged by some that the foramen of a root is usually so small as not to permit the drainage of a blind abscess through it, or the diffusion of medicines through it to the diseased apical tissues. This is undoubtedly the case in a few instances, but clinical observation is conclusive to the contrary in a very large proportion of cases.

In regard to the immediate filling of teeth with blind abscesses, I will quote from the essay already frequently referred to. "Diagnosis of blind abscess of alveolar process is largely guesswork, because the single item of a wet canal does not prove the presence of pus nor the existence of an abscess cavity. When blind abscess exists, the accumulation of pus is so very slight that it will cease altogether after the cause has been removed by closing the apical end of the pulp canal. In nearly all such cases, prompt closure at the apex will be followed by noticeable inflammation, if proper care be observed in manipulation within the pulp canal." It is true that the diagnosis of a chronic and quiescent blind abscess is some times difficult, and we may sometimes treat a pulpless tooth till assured of its healthy condition, and fill it without ever certainly knowing whether a small blind abscess has been cured during the treatment or whether none ever existed, but there are rather numerous instances in which, after removal of the contents of the pulp canal, the discharge of pus is abundantly sufficient to make the diagnosis certain, and most of us have often passed a fine probe through the foramen into acute abscess of this sort and seen it discharge so large a quantity of pus through the pulp canal as very effectually to controvert the statement that "when blind abscess exists, the accumulation of pus is so slight that it will cease altogether after the cause has been removed by closing the apical end of the pulp canal." It must never be forgotten that, though the contents of the pulp chamber and canals were the first cause of the abscess when once established, the contents of the abscess cavity may constitute a continuing cause, which it is as necessary to remove, surgically or therapeutically, as to remove that part of the cause contained in the pulp chamber and canals.

I will quote again: "In all cases the apical end of a pulp canal should be permanently closed as soon as the canal is free from pus or other fluid, and all obstructive matter." I think we can all agree to this if we add the requirment, that by taking sufficient time and giving proper treatment we have ascertained that the canal will remain clean. The statement of the essayist immediately following does not fulfill that requirement, for he says: "I would remove all obstructive matter from the canal, absorb moisture from it till no more appears, and immediately and permanently fill the canal." It is true that a good many blind abscesses will get well after being once pretty thoroughly drained and having the canal permanently closed, but no man can certainly tell which will fail to do so, and it would seem inexcusable to take so great a risk when there is no necessity for it. The cases of blind abscess are those in which immediate root-filling is most dangerous and least justifiable.

In closing the discussion, the essayist quoted from, referring to a remark by Dr. Black to the effect that "Evidence of skill in a dentist is not so much in his success in the large majority of cases that are likely to do well, as with the minority which will give trouble to the patient unless very skilfully handled," said: "Dr. Black has divided immediate root-filling cases into majority and minority, admitting that the large majority are successful. * * * He did not state what the minority is. Close observation for twelve years leads me to estimate the minority at ten per cent. There is no political party under the sun that cares a rap for a minority that represents only ten per cent." This last comparison is an unfortunate one, for in cases of immediate root-filling the minority does not feel bound by the action of the majority, and any man who is obliged to confess to a failure of ten per cent of all his cases of root-filling (for this essayist appears to fill everything

"immediately") must have urgent need to revise his modes of practice in this particular, and if we remember that this ten per cent of failures must be nearly all included in those having putrescent pulp, foul canal or blind abscess, making the proportion of failures probably at least twice or three times as great for those, we hardly need stronger argument against immediate root-filling as a universal practice, than this confession by one of its advocates.

We find a few teeth with dead pulps that are so free from decay as to make it certain that the pulp chambers have never been opened. We do not always feel called on to disturb them, but frequently we do. In most sepsis is plain enough, and they must be treated accordingly. Some appear to be aseptic, but are not really so, and the proportion in which we may be so sure of it as to fill immediately, is rather small.

The immediate filling of teeth with abscess having fistulous opening relates to the disinfection of the pulp chamber and canals, as in all other cases involving sepsis, and to the convenience of using the root canals as channels for the conveyance of force or pressure for the evacuation of the abscess cavities, or the introduction of medicines.

There are many teeth in which the foramina are so small as to be useless for these purposes, and they may as well be closed as soon as disinfected, but there are many others in which the root canal and the fistula furnishing openings at both ends of the abscess cavity and make it possible to give them much more thorough and successful treatment than would be if the canals were closed. This is so much so that a very large proportion of them will get well after one thorough treatment, and many operators feel so sure of that as to fill them at once, but some of them will not get well after one treatment, and it is just as easy to find out whether they will before we fill them as afterward.

Immediate root-filling is theoretically impossible. Even when practicable, if circumstances require it, there are still less in which it is clinically expedient. Many careful and cautious operators, who can control their patients, will seldom practice it. Others, more bold, or whose practice may make it more necessary, will do so very often, but all should draw the line, strictly, at those teeth having the entire pulp canals in a septic condition.

Disinfection of teeth is usually considered to relate only, or at least, chiefly, to the pulp chambers and canals. Occasional appearances seem to indicate instances in which the dentine itself (the contents of the tubes) has become so foul as to require that disinfection reach through its substance. It is usually supposed, I believe, that the tubuli of normal dentine are too small to admit of septic germs, but they certainly do sometimes admit freely the products of decomposition, as is evident by the great change of color. Of course, if the dentine is to be disinfected, the time required is likely to be greater than is necessary to disinfect the chamber and canals. Some work lately done by Dr. Black, which I trust we shall soon hear about, seems to indicate that much organic matter can readily be dried out of an extracted tooth and replaced by water. That diffusibility and displacement are practicable to some extent between the foul contents of dental tubes and fluids or medicines that can be placed in the pulp chamber, is evident from what we often accomplish in the bleaching of teeth. Whether, with the tooth in the mouth, the contents of the tubes can be removed to any great extent by drying, may be doubtful. There is little doubt that the dentine will readily absorb something else to take the place of whatever portion of the tubuli contents can be dried out of them. Clinically, it is evident that in most cases we need not give ourselves concern about the tubuli contents beyond what is accomplished by the disinfectants used with direct reference to the pulp chamber and canals. Of course such medicines must not be coagulants, or we cannot expect them to accomplish anything in the disinfection of dentine.

It has occurred to me, at the last moment, that I might have saved much time by simply warning you of the great difference there is between the great State of Indiana and the equally great State of Illinois. This will more fully appear by a short quotation from a paper, and another from remarks in the discussion following it, both by Dr. J. R. Clayton, at the meeting of the Indiana State Society last June:

"For the past three years my practice has been most radically changed, arguing in the case of pulps purposely destroyed and removed, that the sooner the parts were turned over to nature for rest and restoration the better; that in case of putrescent pulps, if they were giving no trouble while in the canal, none would result when the pulp was removed and the canal cleansed; and, furthermore, if pain had been set up in the peridental membrane, the whole cause was in the putrescence of the pulp, and by removing the cause the effect would cease, and now every pulp canal is cleansed, disinfected, dried and filled at the same sitting, and the success attained has been such that no more thought is given to the future of such operations than to the filling of the plainest molar cavity."

In the discussion he said (in replying to a question about drying roots): "The anterior roots of the lower molars and the buccal roots of the upper molars I do not disturb. I make no effort whatever to get into the buccal roots of the upper molars. I believe I have never seen an abscess on the buccal surface of an upper molar."

In Indiana, therefore, where there are no abscesses on the buccal roots of upper molars, immediate root-filling appears to be a great success, and may, perhaps, be practiced with impunity. But in Illinois such abscesses are very common, and experience and observation have taught us that immediate root-filling must be practiced sparingly and with good judgment. Circumstances must vary treatment.—*Dental Review.*

